# OMIP‐096: A 24‐color flow cytometry panel to identify and characterize CD4+ and CD8+ tissue‐resident T cells in human skin, intestinal, and type II mucosal tissue

**DOI:** 10.1002/cyto.a.24782

**Published:** 2023-09-29

**Authors:** Thomas R. O'Neil, Andrew N. Harman, Anthony L. Cunningham, Najla Nasr, Kirstie M. Bertram

**Affiliations:** ^1^ Centre for Virus Research, The Westmead Institute for Medical Research Westmead Australia; ^2^ The Westmead Clinical School, Faculty of Medicine and Health The University of Sydney Sydney Australia

**Keywords:** CD4+ T cells, CD8+ T cells, enzymatic digestion, flow cytometry, human tissue, tissue‐resident memory T cells

## Abstract

There is a great need to understand human immune cells within tissue, where disease manifests and infection occurs. Tissue‐resident memory T cells (TRMs) were discovered over a decade ago, there is a great need to understand their role in human disease. We developed a 24‐color flow cytometry panel to comprehensively interrogate CD4^+^ and CD8^+^ TRMs isolated from human tissues. When interrogating cells within human tissue, enzymatic methods used to liberate cells from within the tissue can cause cleavage of cell surface markers needed to phenotype these cells. Here we carefully select antibody clones and evaluate the effect of enzymatic digestion on the expression of markers relevant to the identification of T cell residency, as well as markers relevant to the activation and immunoregulation status of these cells. We have designed this panel to be applicable across a range of human tissues including skin, intestine, and type II mucosae such as the vagina.

## 1 BACKGROUND

Tissue‐resident memory cells (TRMs) are an experienced first‐line effector cell defense against reinfection [1, 2, 3]. In chronic HIV infected individuals, CD3^+^CD4^+^ T cells are depleted in intestinal mucosa [4], and cervical CD4^+^ TRMs are a preferentially infected cell target for human immunodeficiency virus (HIV) [5]. In herpes simplex virus infection, mucosal CD4^+^ and CD8^+^ TRMs have been proposed as key targets for vaccines. The ability to accurately identify subsets of T cells within tissues will provide a foundation for understanding the pathology of diseases and assist in the development of therapeutic strategies [1, 3]. We have designed a 24‐color surface phenotyping panel to comprehensively phenotype CD4^+^ and CD8^+^ T cell subsets residing in healthy human mucosal tissues. This panel may also be used on human skin or for comparison with diseased tissues. A challenge to phenotyping cells from tissues is the cleavage of surface markers caused by enzymes used to liberate cells from tissues. We designed this panel with considerations for the cleavage of surface proteins using enzyme‐optimized dissociation methods, and we successfully phenotyped TRMs in human epidermis, dermis, and lamina propria (Table 1).

**TABLE 1:** Summary table for application of OMIP.

Purpose	Characterization of human CD4+ and CD8+ tissue resident T cells isolated from mucosal tissues
Species	human
Cell types	Collagenase digested human tissue: human skin, intestine, type II mucosa (e.g., vagina, cervix)
Cross‐reference	OMIP‐070, OMIP‐082

There are three types of tissue that make up the anorectal and genital tracts: skin, type I mucosa, and type II mucosa. Skin is made up of an outer epidermal layer, including a superficial impervious keratinized layer of dead cells (stratum corneum), and an underlying dermal layer separated by a basement membrane. Skin covers the outermost parts of the body. Type II mucosae, such as vagina and inner foreskin, have an epidermal layer and an underlying lamina propria, a structure similar to the dermis, and contains no or a minimal keratinized layer. Type II mucosa is often found between outer skin and type I mucosal surfaces. Type I mucosae, such as endo‐cervix and colorectal tissues, have a single layer of columnar epithelium, with underlying lamina propria.

TRMs require enzymes to liberate them from tissues. For skin and type II mucosa, dispase breaks down the basement membrane between epidermal and dermal/lamina propria layers, allowing these layers to be independently studied. Collagenase is generally a blend of several enzymes that will break down collagen, freeing the cells from the tissue. Unfortunately, enzymatic digestion procedures are known for cleaving cell surface proteins on immune cells [6]. Some studies will rest their cells for a period of time to recover this protein expression [7], although this has been shown to not truly reflect the cells as they were in tissue [8]. This non‐specific cleavage of markers requires a series of optimization steps prior to panel development to be confident that the cells that are acquired are similar to those (in situ) within the tissue. A significant novelty of this publication is the application of our optimization protocols that minimize the effects of enzymatic cleavage on surface proteins [6, 9, 10]. This is primarily performed through the identification of the affected markers, and testing multiple antibody clones and alternative enzymes (Figures S3 and S4).

**FIGURE 1:**
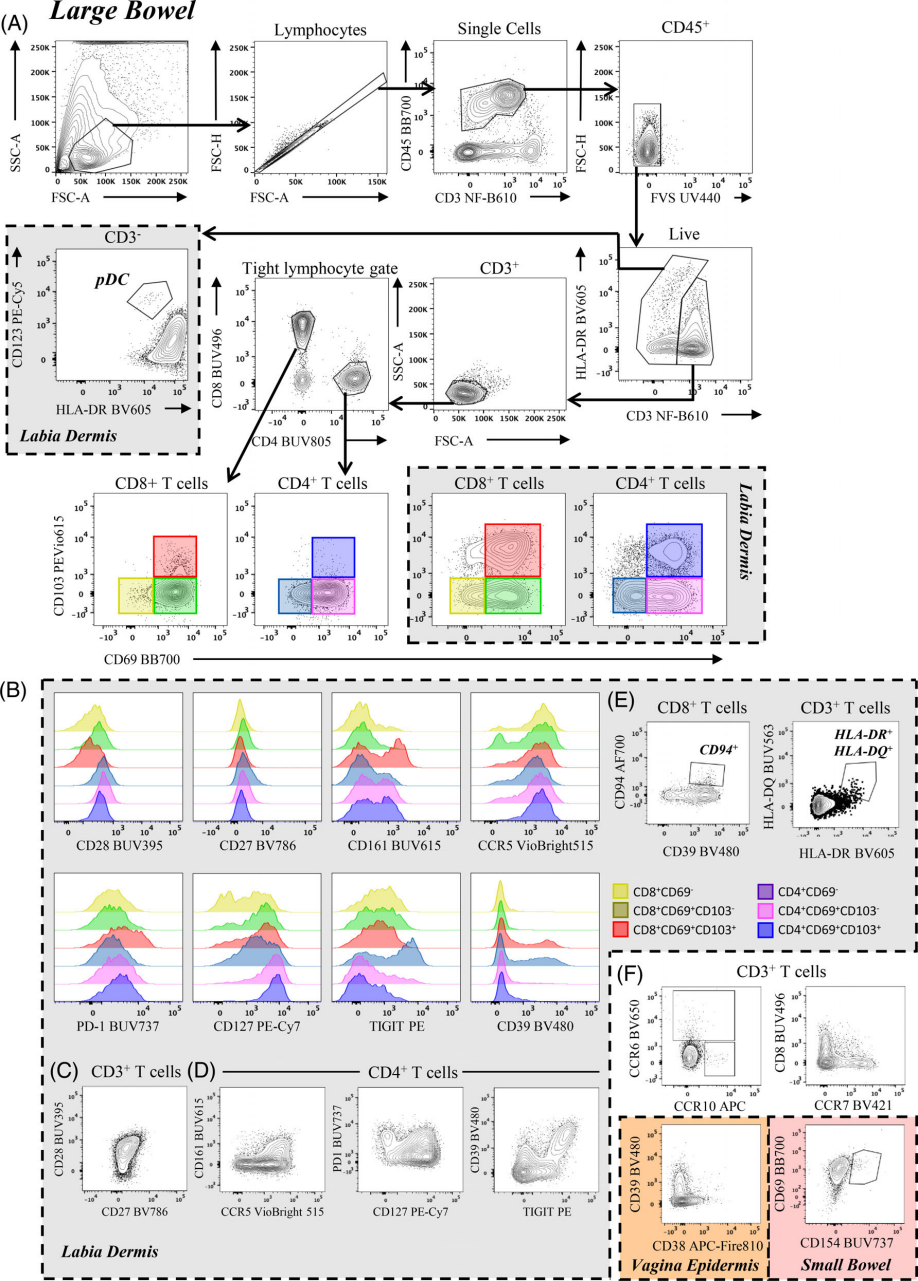
Gating strategy to identify CD3+ TRM cells in human anogenital tissues. Cells isolated from healthy human large bowel were stained and acquired on the BD FACSymphony A5 instrument. Cells were gated on single cells (FSC‐A/FSC‐H), CD45^+^ lymphocytes (CD45/CD3) and live cells (FVS UV440/FSC‐H). HLA‐DR is used to gate on CD3^+^ T cells, to account for the bright HLA‐DR^+^ autofluorescent mononuclear phagocyte cells spreading into the CD3 channel. On CD3^−^ cells, pDCs are identified using CD123 and HLA‐DR. On CD3^+^ T cells, an additional tight gate is drawn around the lymphocyte cluster (FSC‐A/SSC‐A) to remove any additional contaminating mononuclear phagocytes. CD4^+^ T cells and CD8^+^ T cells are gated separately, and TRM subsets are gated on using CD69 and CD103 (non‐TRM: CD69^−^CD103^−^; CD103^−^ TRM: CD69^+^CD103^−^; CD103^+^ TRM: CD69^+^CD103^+^). Labia dermal cells are included to compare CD103^+^ TRMs in different tissues. (B) Histograms of markers expressed by labia dermal TRM subsets, split into CD4^+^ and CD8^+^ subsets. (C, D) Alternative representative FACS plots of those markers are presented as a histogram (E, F) Other marker expressions are represented using a variety of tissues where expression is prominent. (E) Representative expressions of CD94 on CD3^+^CD8^+^ cells and HLA‐DQ on CD3^+^ HLA‐DR^+^ T cells from labia. (F) Representative images of CCR6, CCR10, and CCR7 expression on CD3^+^ T cells from the large bowel, CD38 expression on CD3^+^ T cells from the vagina epidermis, and CD154 expression on CD3^+^ T cells from the small bowel. Representative figures are color‐coordinated: large bowel = white, labia dermis = grey, vagina epidermis = orange, small bowel = light‐red. [Color figure can be viewed at wileyonlinelibrary.com]

We aimed to identify both CD4^+^ and CD8^+^ TRMs (Figure 1). Markers associated with residency span homing (chemokine) receptors, those associated with cell maintenance in tissue, and those that differ from circulating and non‐resident tissue cells [11]. The best characterized receptors are CD69 and CD103, which delineate three main populations: CD69^−^CD103^−^ non‐TRMs, and two CD69^+^ TRM populations delineated by CD103 expression. Higher proportions of CD103^+^ cells are found in CD8^+^ TRMs, but it is expressed on both CD4^+^ and CD8^+^ T cells. CD103^+^ TRMs have functional and migratory differences to the CD103^−^ counterpart [12, 13]. There can be large differences in expression of CD103 between tissues demonstrated in Figure 1 (large bowel mucosae and labia dermis). A small cluster of CD69^−^CD103^+^ cells has been described as a TRM re‐entering circulation [14]. These cells exit through lymphatics, and so the lymph‐homing marker CCR7 was included to identify these cells. Markers associated with T cell tissue homing (CCR5, CCR6 [15]) and residency (CD127 [16], PD1 [11]) were also included.

In early studies, TRMs were thought to be at the late stage of differentiation based on surface expressions. Recent studies suggest that TRMs can recirculate (CD4^+^ TRMs in humans [14]; CD8^+^ TRMs in mice: [17]) and, further, that they can revert to a central memory phenotype [17]. We have therefore included conventional memory markers (CD27, CD28, CCR7) to capture the memory T cell phenotype. In both CD4^+^ and CD8^+^ T cells, CD27 is used to delineate early differentiation (naïve > central memory > transitional memory) from later subsets (effector > terminally differentiated memory). In blood, expression of CCR7, a lymph‐homing molecule, describes a TCM or naïve cell. CD28 is a co‐stimulatory molecule, which is upregulated following antigen stimulation and is decreased during T cell exhaustion. Therefore, together, we can identify a naïve cell (CCR7^+^CD27^+^CD28^lo^), central memory (CCR7^+^CD27^+^CD28^hi^), transitional memory (CCR7^−^CD27^+^CD28^hi^), effector memory (CCR7^−^CD27^−^CD28^hi^) and a terminally differentiated memory cell (CCR7^−^CD27^−^CD28^−^) in both CD4^+^ and CD8^+^ T cells.

We have also included two markers associated with the regulatory function of T cells: CD39 and TIGIT. CD39 is an exhaustion marker on virus‐specific CD8^+^ T cells in chronic diseases [18] and can also disrupt the anti‐inflammatory responses [19]. CD8^+^CD39^high^ TRMs were recently associated with positive prognosis of breast cancer [20]. In another study, regulatory T cells that were CD39^high^ remain more stable under inflammatory conditions, compared to CD39^low^ Tregs which lose their FOXP3 expression [21]. TIGIT expression in CD4^+^ T cells is associated with thymic regulatory T cells [22]. A recent study found a TIGIT^+^CD39^+^ CD4^+^ TRM subset enriched in mucosa in Crohn's disease [23].

On T cells, CD161 describes unique subsets in CD4^+^ and CD8^+^ T cells. In CD8^+^ T cells, CD161 expression defines a cytotoxic cell, and may play a role in CD8^+^ T cell homing to tissue [24]. In CD4^+^ T cells, CD161, with CCR6 and CCR10 describe effector T cell subsets. Th17 and Th22 cells play an important role in tissue maintenance, have been implicated in HIV infection and transmission, and their dysregulation can affect the severity of autoimmune diseases. CD161 and CCR6 are identifying markers for Th17 cells, while CCR6^−^CCR10^+^ identifies Th22 cells. We did not include markers for identifying other CD4+ T cell subsets as their definition within tissue remains unsubstantiated and are not the primary focus of the panel.

In CD8^+^ T cells, CD94, a C‐type lectin receptor thought to have a regulatory function [25], is found on tumor‐infiltrating cells and has been found on a unique cluster of CD8^+^ T cells in Crohn's affected bowel tissue [23, 26], although a clear definition of its function on T cells remains largely elusive.

The study of T cell activation has applications across a range of disciplines, such as virology, autoimmune, transplant immunology, among others. We have included a range of activation markers to define early‐ and late‐activation specifically chosen for tissue‐derived cells. HLA‐DR is a late‐activation marker, often expressed at low levels on activated T cells compared to mononuclear phagocytes. Another late activation marker, HLA‐DQ, was brought to our attention by its differential expression in single‐cell RNA sequencing data (Figure S8; [27, 28, 29]). CD154 (CD40LG) binds CD40 on antigen presenting cells and is required for Th17 polarization [30]. It is an early activation marker that was differentially expressed in transcriptomic analysis and played a role in Crohn's disease [23]. CD38, an early activation marker, is involved in cell adhesion and signal transduction [31]. Due to donor‐to‐donor variability, low, mild, or high amounts of inflammation may markedly influence proportions and expressions of activated cells. We are able to show low expressions of CD154 (small bowel) and CD38 (vagina epidermis) owing to the likely inflamed status of those individual tissues (Figure 1). CD123 was also included to identify and measure the presence of plasmacytoid dendritic (pDC) cells, which accumulate in inflamed [32] or virally infected tissue [33]. All reagents are summarised in Table 2.

**TABLE 2:** Reagents used for OMIP.

Specificity	Fluorochrome	Clone	Purpose
CD45	BB790	HI30	Leukocytes
CD3	NovaFluor B610‐70s	UCHT1	T lymphocyte
Viability	FVS UV440		Viability
HLA‐DR	BV605	G46‐6	Gate out MNPs; Late Activation on T cell
CD123	PE‐CY5	6H6	Plasmacytoid Dendritic Cell
CD4	BUV805	OKT4	Helper T cell
CD8	BUV496	SK1	Cytotoxic T cell
CD94	AF700	KLRD1	Subset: Functional marker of T cells
CD69	BB700	FN50	TRM subset
CD103	PEVio615	REA803	TRM subset
CD127	PE‐CY7	R34.34	TRM; tissue homeostasis
PD‐1	BV711	EH12.1	TRM
CCR7	BV421	2‐L1‐A	Naïve; TCM; Lymph homing
CD27	BV786	L128	Memory: TCM
CD28	BUV395	CD28.2	Memory: differentiation
CD161	BUV615	HP3G10	CD4: Th17; CD8: cytotoxicity
CCR6	BV650	11A9	Subset: Th17; Tissue homing
CCR10	APC	1B5	Subset: Th22
CD39	BV480	TU66	Immunoregulation
TIGIT	PE	A15153G	Immunoregulation
CCR5	VioB515	REA245	Tissue homeostasis; HIV entry
HLA‐DQ	BUV563	TU169	Late Activation; Differentially expressed in RNA‐seq
CD38	APC‐Fire810	HIT2	Early Activation
CD154	BUV737	TRAP1	Early Activation

## 2 Similarities to other OMIPs

Many OMIPs have focused on phenotyping human T cell subsets and activation, though all are in the context of blood‐derived lymphocytes. Particularly, OMIP‐071 extensively covers both the memory and effector T cell subsets of both CD4^+^ and CD8^+^ T cells. However, they primarily use chemokine receptors for the identification of effector subsets. In tissue, using chemokine receptors to identify helper T cell subsets are somewhat confounded, as they are more often described as markers for homing to specific tissues; CCR6 to gut, CXCR6 to lung, and so forth. Further, the use of chemokine receptors in tissues would require careful curation, as they are particularly prone to enzymatic cleavage. OMIP‐082 and ‐070 have designed panels for use in enzymatically liberated human tissue cells; the panel presented in OMIP‐070 was designed for the identification of NK cells, whereby CD56 was consistently cleaved and fortunately resolved by using an alternative target, NKp46; they did not test alternative enzyme products, as was performed in OMIP‐82 and in this OMIP. The panel design in OMIP‐82 is focused on identifying innate lymphoid cells, NK cells, MAIT cells, and gamma‐delta T cells in gastrointestinal tissues, which does not require dispase digestion to investigate cells in epidermal and dermal tissues. Currently, there is no OMIP that optimizes the combination of enzyme‐digestion combined with antibody‐clone selection to identify TRMs from both the epidermis and dermis of human tissues.

## AUTHOR CONTRIBUTIONS

**Kirstie Melissa Bertram:** Investigation; conceptualization; writing – review and editing; methodology; validation; formal analysis; visualization; supervision; data curation. **Thomas O’Neil R:** Conceptualization; investigation; methodology; validation; visualization; writing – review and editing; formal analysis; data curation; writing – original draft. **Andrew N Harman:** Writing – review and editing; supervision; funding acquisition; resources; project administration. **Anthony L. Cunningham:** Funding acquisition; writing – review and editing; supervision; resources; project administration. **Najla Nasr:** Funding acquisition; writing – review and editing; supervision; resources; project administration; conceptualization.

## FUNDING INFORMATION

This work was supported by the Neil and Norma Hill Foundation, the Westmead Medical Research Foundation, and the Australian National Health and Medical Research Council (NHMRC ideas grant #APP1181482).

## CONFLICT OF INTEREST STATEMENT

The authors declare that the research was conducted in the absence of any commercial or financial relationships that could be construed as a potential conflict of interest.

## Supporting information


**Data S1.** Supporting information.

**Data S2.** Supporting information.

**MIFlowCyt** MIFlowCyt item checklist.
