# Supplementary material for: OMIP‐096: A 24‐color flow cytometry panel to identify and characterize CD4+ and CD8+ tissue‐resident T cells in human skin, intestinal, and type II mucosal tissue
Source: Cytometry A. 2023 Sep 29;103(11):851–6. doi: 10.1002/cyto.a.24782 (PMC10953338; doi:10.1002/cyto.a.24782)
Supplement: Supplementary file 1 — Data S1. Supporting information. [file CYTO-103-851-s002.pdf]

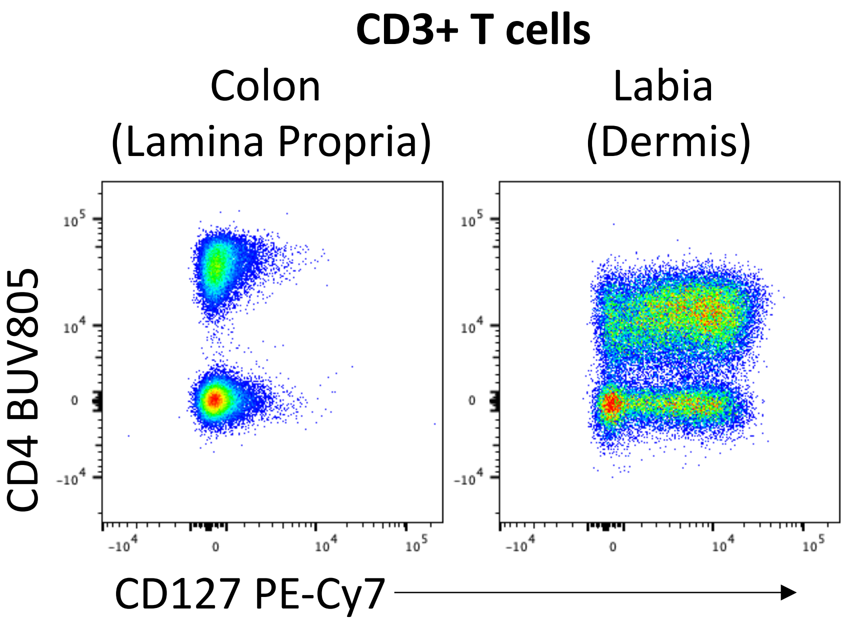
CD3+ T cells
Colon
(Lamina Propria)
Labia
(Dermis)
CD4 BUV805
CD127 PE-Cy7

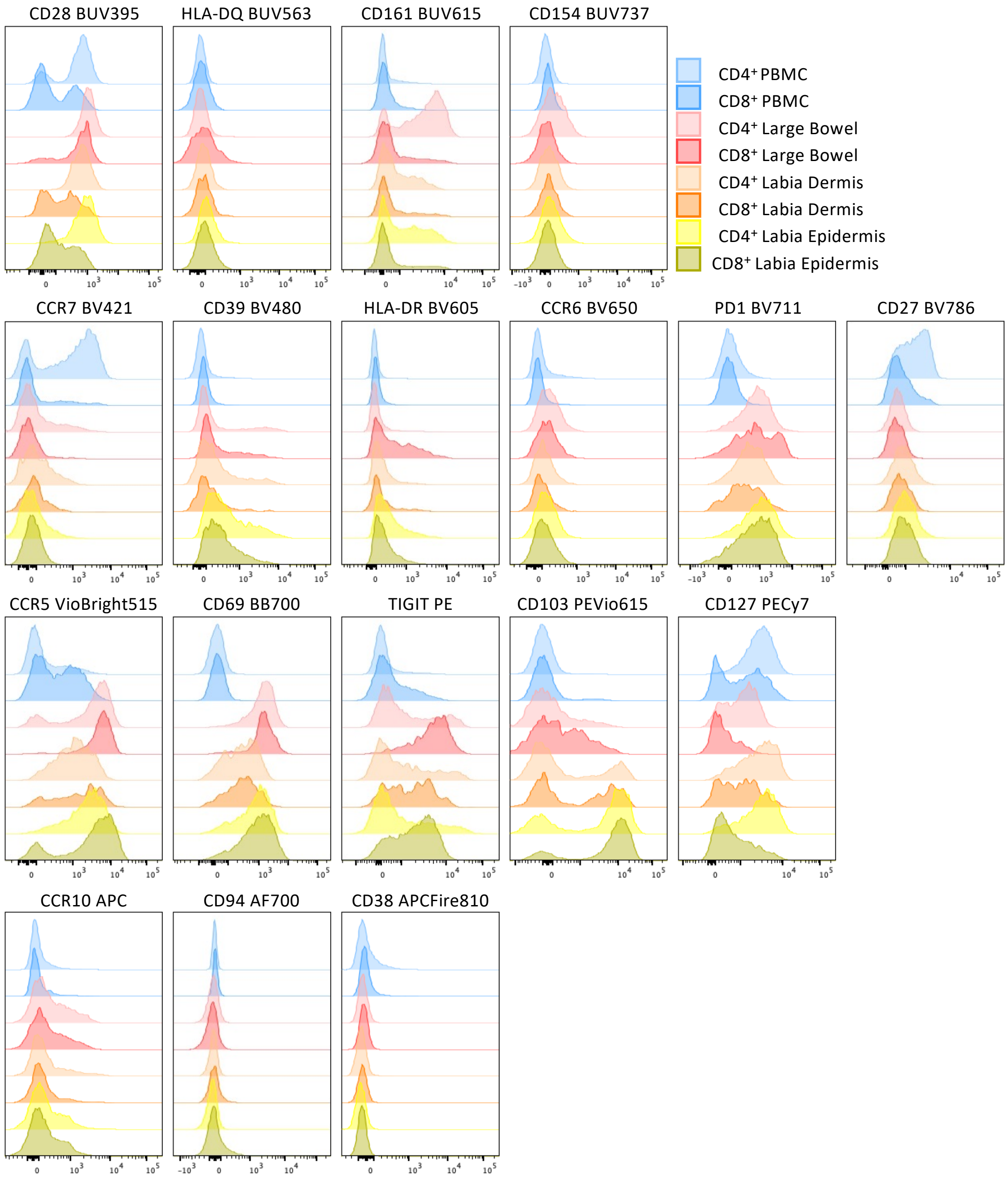
CD28 BUV395
HLA-DQ BUV563
CD161 BUV615
CD154 BUV737
CD4⁺ PBMC
CD8⁺ PBMC
CD4⁺ Large Bowel
CD8⁺ Large Bowel
CD4⁺ Labia Dermis
CD8⁺ Labia Dermis
CD4⁺ Labia Epidermis
CD8⁺ Labia Epidermis
CCR7 BV421
CD39 BV480
HLA-DR BV605
CCR6 BV650
PD1 BV711
CD27 BV786
CCR5 VioBright515
CD69 BB700
TIGIT PE
CD103 PEVio615
CD127 PECy7
CCR10 APC
CD94 AF700
CD38 APCFire810

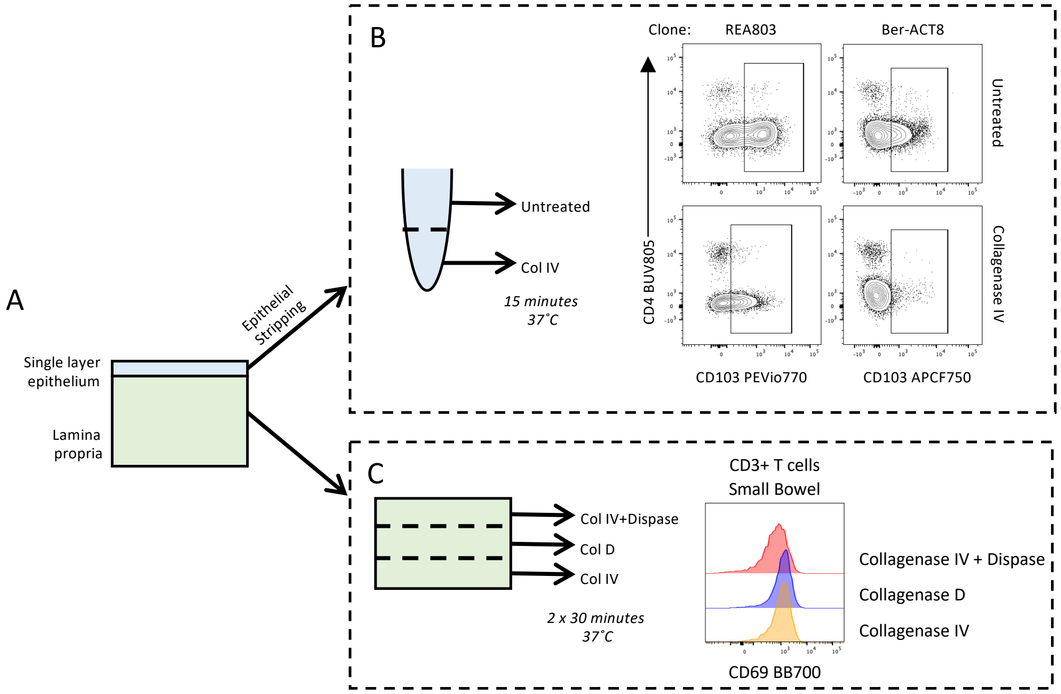
A
Single layer epithelium
Lamina propria
Epithelial Stripping
B
Untreated
Col IV
15 minutes 37˚C
Clone: REA803 Ber-ACT8
CD4 BUV805
Untreated
Collagenase IV
CD103 PEVio770
CD103 APCF750
C
Col IV+Dispase
Col D
Col IV
2 x 30 minutes 37˚C
CD3+ T cells Small Bowel
Collagenase IV + Dispase
Collagenase D
Collagenase IV
CD69 BB700

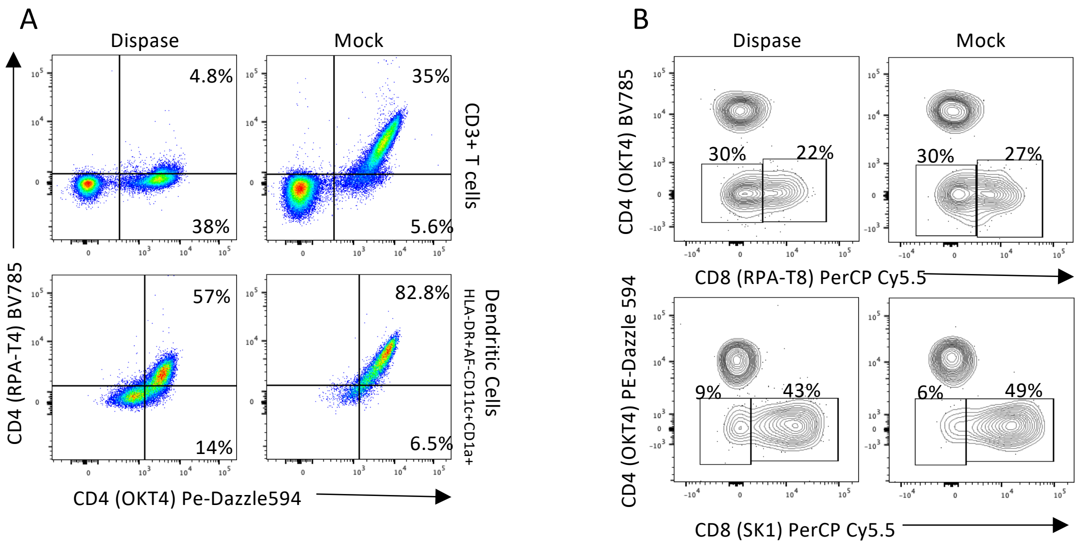

A
Dispase
Mock
4.8%
38%
35%
5.6%
CD3+ T cells
57%
14%
82.8%
6.5%
Dendritic Cells
HLA-DR+AF-CD11c+CD1a+
CD4 (RPA-T4) BV785
CD4 (OKT4) Pe-Dazzle594
B
Dispase
Mock
CD4 (OKT4) BV785
30%
22%
30%
27%
CD8 (RPA-T8) PerCP Cy5.5
CD4 (OKT4) PE-Dazzle 594
9%
43%
6%
49%
CD8 (SK1) PerCP Cy5.5

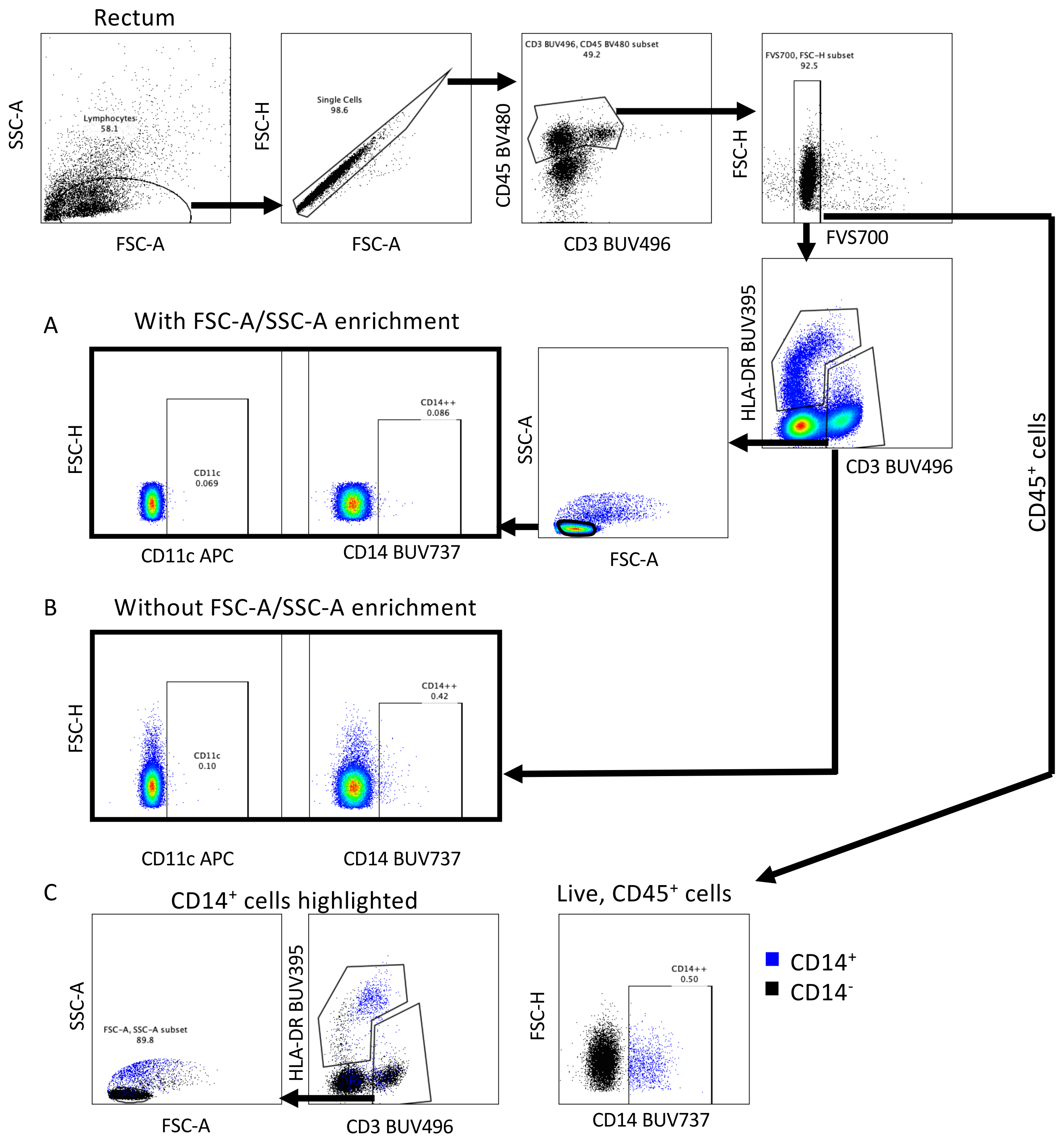

Rectum
SSC-A
FSC-A
Lymphocytes
58.1
FSC-H
Single Cells
98.6
CD3 BUV496, CD45 BV480 subset
49.2
CD45 BV480
CD3 BUV496
FVS700, FSC-H subset
92.5
FVS700
HLA-DR BUV395
CD45+ cells
A
With FSC-A/SSC-A enrichment
CD11c
0.069
CD14++
0.086
CD11c APC
CD14 BUV737
B
Without FSC-A/SSC-A enrichment
CD11c
0.10
CD14++
0.42
C
CD14+ cells highlighted
FSC-A, SSC-A subset
89.8
Live, CD45+ cells
CD14++
0.50
CD14+
CD14-

A.

B.

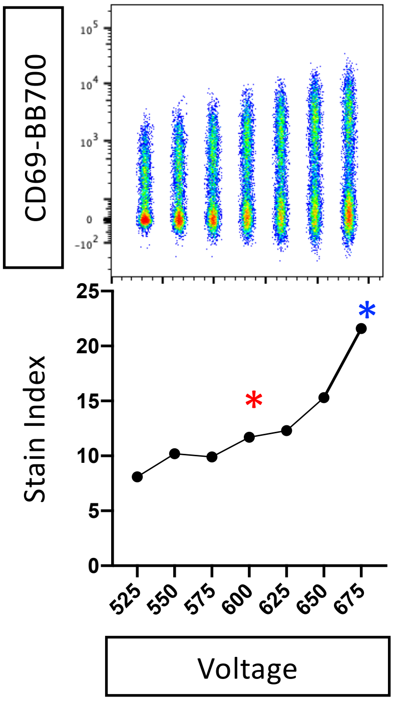

CD69-BB700
10^5
10^4
10^3
0
-10^2
Stain Index
25
20
15
10
5
0
525
550
575
600
625
650
675
Voltage

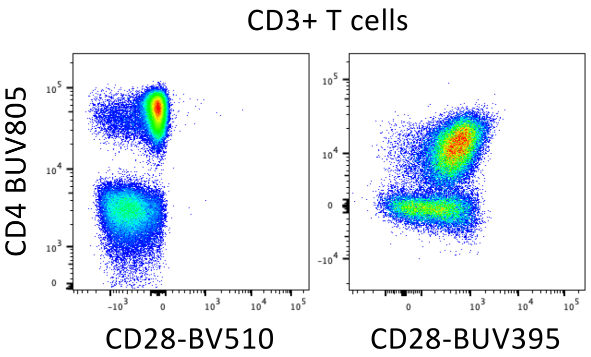
CD3+ T cells
CD4 BUV805
CD28-BV510
CD28-BUV395

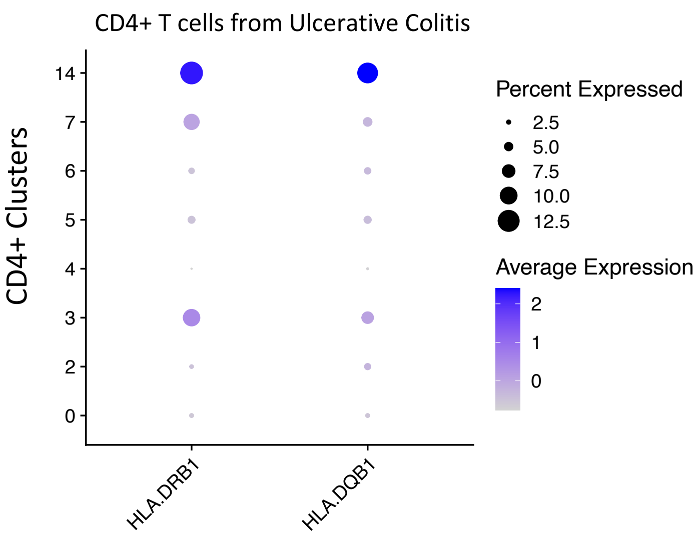

Boland et al., 2020

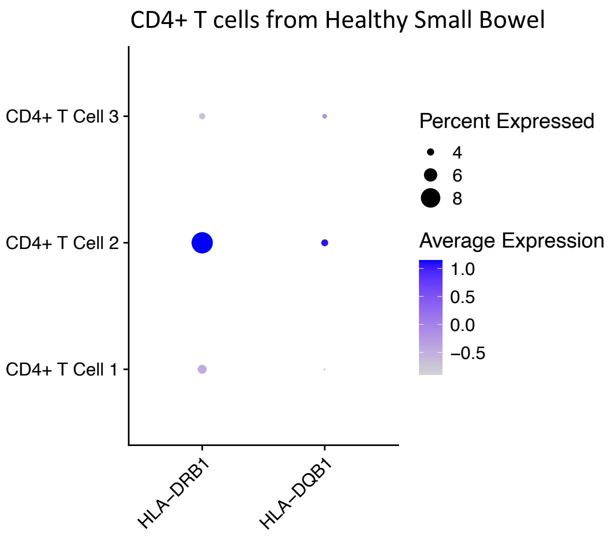

Gut Cell Atlas
Martin et al., 2019

A) PBMC Titrations

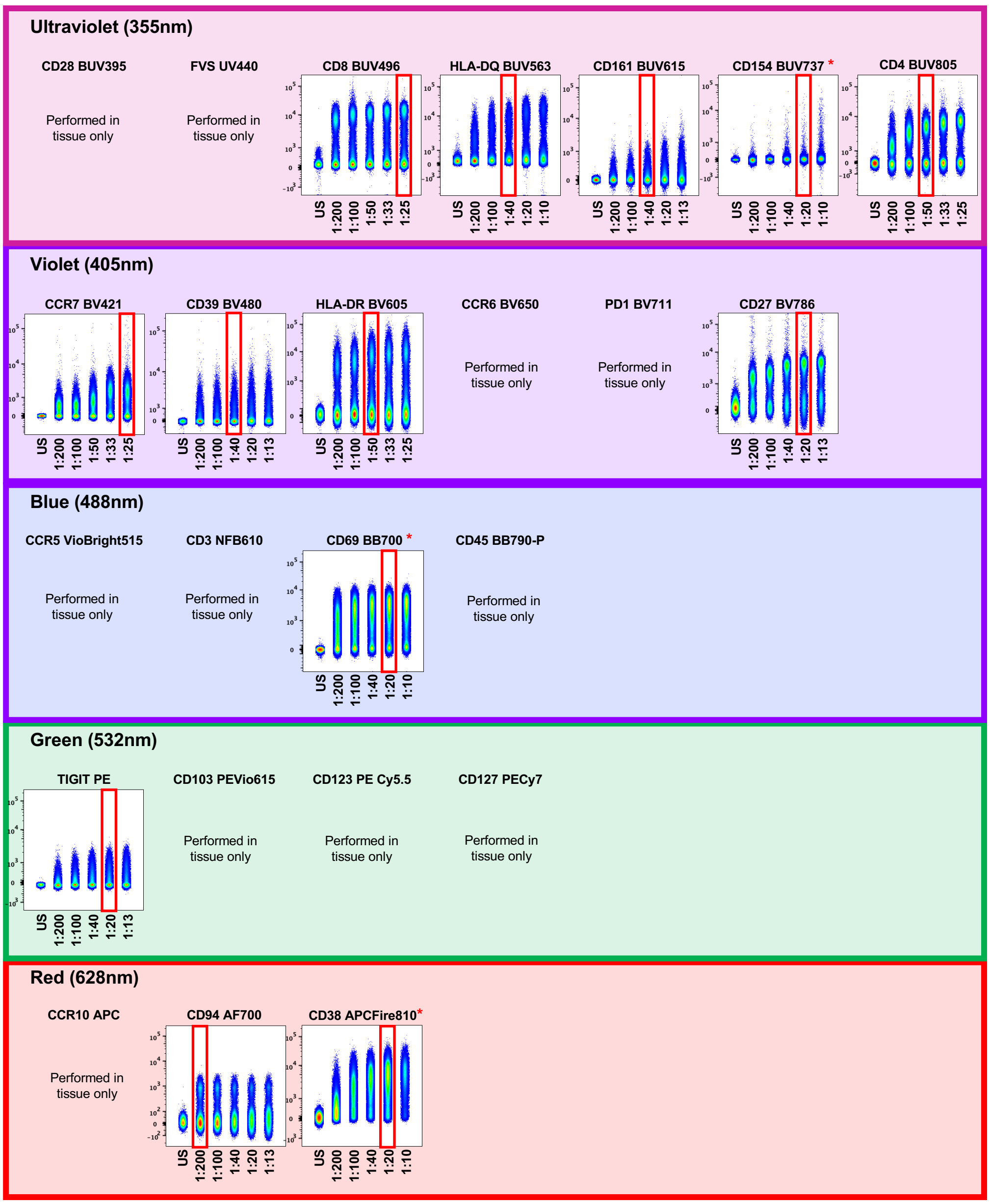

* Activated with PHA + IL-2 for 8 hours

us = unstained

B) Tissue Titrations

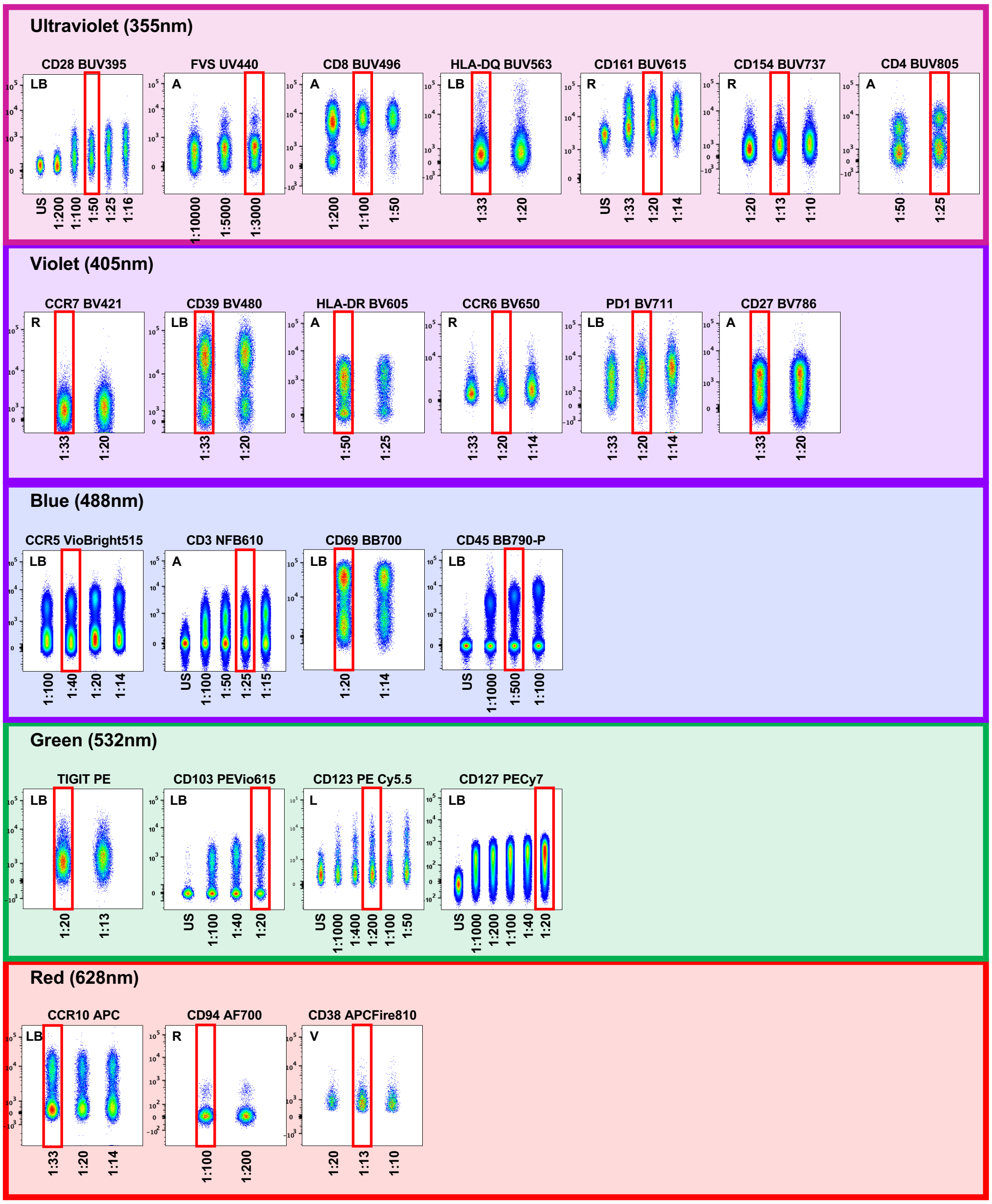

A = Abdomen
LB = Large Bowel
R = Rectum
L = Labia
V = Vagina
US = unstained
