# Supplementary material for: OMIP‐096: A 24‐color flow cytometry panel to identify and characterize CD4+ and CD8+ tissue‐resident T cells in human skin, intestinal, and type II mucosal tissue
Source: Cytometry A. 2023 Sep 29;103(11):851–6. doi: 10.1002/cyto.a.24782 (PMC10953338; doi:10.1002/cyto.a.24782)
Supplement: Supplementary file 2 — Data S2. Supporting information. [file CYTO-103-851-s003.docx]

**Purpose**

This 24-colour panel will identify and characterise tissue resident CD3^+^ T cells. It is developed for the FACSymphony A5 cytometer (8 lasers and 46 detectors: Online Table 1). Specifically, this panel can be used to comprehensively phenotype CD4^+^ and CD8^+^ T cell subsets residing in healthy human tissues (skin, type I and type II mucosa) using our previously optimised digestion protocols (DOI: 10.3389/fimmu.2021.727952; 10.1189/jlb.4A1116-496R). Furthermore, this panel can be used to assess activation markers on T cells in diseased tissues. Our panel is suitable for the study of tissue-derived CD4 and CD8 T cells as it addresses the consequential cleavage of CD3 T cell surface markers following enzymatic liberation from tissues in addition to the choice of antibody clones and enzyme types, titration and voltage optimisations.

**Definitions:**

Primary, Secondary and Tertiary marker expressions are well defined categorisations described by Mahnke & Roederer (2007) (DOI: 10.1016/j.cll.2007.05.002)

- **Primary marker expression:** Clear resolution between positive and negative, with binary on/off expression, such as CD45, CD3, CD4.
- **Secondary marker expression:** A marker that has a continuum of expression, often dense. Resolution may be more difficult, and a second marker may be used to identify the population (e.g. CD103 helps draw out a positive CD69^+^ or CD127^+^ gate).
- **Tertiary marker expression:** Tertiary markers are either lowly expressed markers, or markers that are context-dependent, such as those dependent on inflammation/activation or tissue-type. Therefore, a lot of activation markers, like CD154 and CD38, are tertiary. “**Tertiary/Secondary**” is used throughout to describe a highly variable marker; variability between tissues, activation, or markers such as CD27, which are high in blood and low in tissue.

Other definitions:

- **Enzymatic cleavage:** A decrease in a surface marker expression due to enzymes that non-specifically cleave antibody-binding sites. Primarily, the fluorescence intensity is assessed, with considerations to percentage for secondary or tertiary markers with difficult resolution.
- **No confounding Spillover Spreading Error (SSE) issues:** is stated when an optimally selected clone of antibody is titrated and voltrated, and is observed to have a clear resolution with no spectral spill-over occurring after compensation.

**Tissue sizes and cell yields**

The tissue explants used to optimise this panel range from 10cm^2^ (e.g. 2cm x 5cm labia or vagina) to 560cm^2^ (e.g. A4-paper sized abdomen). We used the underlying mucosa (dermis and lamina propria) which may yield between 5 to 20 x 10^6 total cells. The panel may be stained on small biopsies, which have yielded 1-4 x 10^6^ total cells (DOI: 10.3389/fimmu.2021.727952**).**

**Assessing enzymatic Cleavage**

The liberation of cells from tissues requires the use of enzymes which can cleave cell surface proteins. In our research, we used two enzymes: **Dispase** digests the basement membrane, allowing the separation of epidermis from underlying mucosa, and **Collagenase** to break down collagen and liberate the cells from the solid tissue. With this approach, two considerations were raised. The first was how we were to have a no-enzyme control when the enzyme itself is necessary to liberate the cells. For this, we used lymphocytes from the epithelium of gut tissue. These cells do not require enzymes for liberation and express Tissue Resident Memory T cell (TRM) Markers, such as CD69 and CD103. The second consideration was to optimise the panel using cells derived from tissues where the marker of interest is expressed. For example, CD127 is variably expressed between tissues (Online Figure 1). We avoided using PBMCs to titrate some antibodies as the expression and/or densities of markers will differ between blood and tissue T cells (DOI: 10.1016/j.immuni.2016.07.007) (Online Figure 2). Further, exposing PBMCs in suspension to enzymes without the tissue architecture is a harsh process compared to liberation of cells residing in tissues and which are shielded by the tissue environment. It is for this specific reason that our digestion protocols limit the cell exposure to enzymes to 2 hours maximum, as liberated cells from tissues will be exposed to these enzymes.

Clone testing:

When we optimise an antibody that we have not previously used in tissue, we compare commonly used clones from the literature, such as the CD4 clones: OKT4 and RPA-T4. Clone optimisations are often focused on secondary and tertiary markers (e.g. CD69). Primary markers such as CD45 and CD3 are more binary, and their cleavage is much more evident (e.g. CD4, Online Figure 4).

To assess whether collagenase cleaves cell surface markers, we used a tissue that does not require enzymatic liberation and treated it with enzymes. This has been done previously by others using spleen tissue from mice (DOI: 10.1556/EuJMI.2.2012.2.3). Here we have used the stripped epithelial layer of gut. We know that the CD8^+^ T cells found in intraepithelial gut tissue are TRMs and are proportionally high for CD103 expression. Stripped tissue was treated for 15 minutes with Collagenase IV or Collagenase D (200U/mL) and stained with either CD103 *REA803* PEVio770 or CD103 *Ber-ACT8* APCFire750 (Online Figure 3B). The *REA803* clone on PEVio770 had clear resolution without Collagenase on CD3^+^ T cells. For the *REA803* clone, there was a noticeable reduction in fluorescence intensity, though detection of a positive population was still possible. The *Ber-ACT8* clone in untreated cells had a lower fluorescence intensity, which was further reduced when treated with collagenase. As mentioned above, we wouldn’t normally treat cells already in suspension with enzymes, as it is excessively harsh to the cell surface. We consistently detect a better resolution using the *REA803* clone on cells isolated directly from tissues using the final optimised digestion protocol (gating strategy; described below).

Next we assessed whether dispase cleaves cell surface markers chosen for this panel. We found CD4 and CD8 to be severely affected (Online Figure 4). The CD4 *RPA-T4* clone binds the D1 region of CD4 (DOI: 10.1038/icb.2014.102) and the CD4 *OKT4* clone binds the D3 region (DOI: 10.1084/jem.172.4.1233), and they do not compete for binding. When tissue had been treated with dispase prior to collagenase, we found substantial reduction in CD4 *RPA-T4* expression, in contrast to CD4 *OKT4* (Online Figure 4A). However, we found that CD4 expression on dendritic cells was not as substantially reduced. This is important to note, as CD4 expression will impact the outcome of downstream experiments investigating HIV. HIV is known to bind to the same domain as the *RPA-T4* clone (DOI: 10.1089/088922299309702; DOI: 10.1084/jem.172.4.1233). Therefore, we cannot use dispase-treated CD4^+^ T cells in HIV infection assays, as the HIV binding site will be cleaved. Interestingly, dispase treatment does not affect dendritic cells to the same degree. CD8 *RPA-T8* on T cells is substantially cleaved compared to CD8 *SK1*, which appears partially cleaved (Online Figure 4B). The effects of enzymatic cleavage was assessed across the whole panel.

Enzyme testing:

To assess whether different **collagenase** types cleave cell surface markers, we treated the gut tissue with either Collagenase D or Collagenase IV. To measure the partial cleavage effects of **dispase** on the optimised panel, we treated the same gut tissue with Collagenase IV or with a blend of Collagenase IV + dispase. CD69 expression on CD3^+^ T cells was slightly reduced by Collagenase IV compared to Collagenase D, and further reduced in the presence of Dispase (Online Figure 3C). Collagenase IV was chosen going forward, as a) collagenase IV liberates more cells (11.7 x 10^6) than collagenase D (2.7 x 10^6) from the same tissue, and b) resolution of CD69^+^ T cells was maintained by using CD103 as a co-expressed marker (gating strategy). The cleavage effects of different enzymes have been previously demonstrated in OMIP-082.

In summary, optimising a flow cytometry panel to isolate cells from tissue requires the correct mix of antibody clones, enzyme types, lots and batches with specific consideration to the subset of cells one intends to investigate. For this panel, we found Collagenase IV and dispase to be optimal. However, it’s important to note that individual studies need to repeat optimisations, as enzymes are not entirely compatible between batches and lots. For example, a study by Pena-Cruz (DOI: 10.1172/JCI98943) used dispase to separate vaginal epithelium from lamina propria, and Collagenase IV for cell liberation, and did not find a severe reduction in CD4 *RPA-T4* nor CD8 *RPA-T8* that we demonstrate here and have seen consistently.

**Panel Design**

We started by considering the placements of CD127 and CD103 as we needed specific clones to minimise the effects of enzymatic cleavage, and because of limitations in commercial fluorophore availability. CD127 is a secondary marker, with medium-low brightness and is variable between tissues (Online Figure 1). The optimal clone, *R34.34*, was available through R&D on PE-Cy7 and showed great resolution between positive and negative population. The optimisation of CD127 had been previously demonstrated in OMIP-082 (DOI: 10.1002/cyto.a.24529). There was potential for spill-over spreading error (SSE) with the B820 channel (CD45). Due to relatively low expression after optimising the titre and voltage, we saw no problems between PE-Cy7 and B820. CD103 is a crucial marker for identifying a subset of resident T cells. CD103 (clone: *Ber-ACT8*) was first optimised conjugated to fluorophore APC Fire750 (Online Table 2). However, during test runs of the panel, it was found to be inconsistent due to enzymatic cleavage. Following optimisations and the formal testing described above, the *REA803* clone of CD103 was found to have better resolution (Online Figure 3B) and was moved to PE-Vio615 due to conjugate availability. We did not find issues with the potential SSE into B610 (CD3).

To identify live CD4^+^ and CD8^+^ T cells, a viability stain, CD45, HLA-DR, CD3, CD4 and CD8 were assigned to the panel. While HLA-DR is a secondary/tertiary marker on T cells, all other markers are primary. The placement of these markers is critical to minimise downstream spillover effects. One additional consideration is that we were limited to the fluorochrome commercial availability of certain clones, such as CD4 (clone: *OKT4*) and CD8 (clone: *SK1*; Online Figure 4). CD45 was bought as a custom on BB790-P to make use of a regularly unused detector (B820). The potential SSE into V820 (CD27) was alleviated by lowering the CD45 titre as low as possible (from 0.5uL to 0.15uL; DOI: 10.1002/cyto.a.24529), maintaining separation whilst minimising SSE. UV440 was used as fixable viability stain. A clear resolution was achieved and there were no confounding SSE. The relatively dim fluorophore BUV395 was reserved for CD3, where clear resolution was easily obtained. Because of later changes to the panel (Online Table 2), CD3 was moved from BUV395 to NovaFluor B610-70S (NFB610), which proved to have better resolution when specifically placed against HLA-DR (BV605). By gating on HLA-DR^low^, we removed mononuclear phagocytes (MNPs) or autofluorescent macrophages that were spilling into B610 (gating strategy). Here the HLA-DR^hi^ cells appear to be positive for CD3, however this is due to autofluorescence from macrophages in this emission range (DOI: 10.3389/fimmu.2021.727952), so the gate must be placed to exclude the HLA-DR^hi^ autofluorescent cells . NFB610 has potential SSE into R670 (CCR10), V610 (HLA-DR) and V670 (CCR6). However, since CD3 is a primary marker, we can keep its brightness low while maintaining adequate resolution, and therefore reducing SSE into the affected channels. For CD4 and CD8, we needed specific antibody clones (*OKT4* and *SK1*, respectively; Online Figure 4). CD8 was assigned to the dim BUV496 and CD4 to a low/mid brightness fluorophore BUV805. There was potential for SSE from BUV496 into channels U586 (HLA-DQ) and Y586 (TIGIT), but we found no issues. There were no confounding SSE expected or observed from BUV805. HLA-DR has a bright basal expression on MNPs, but a comparatively low expression on T cells. For this reason, a dim/medium fluorophore (BV605) was chosen over a dim fluorophore (what we regularly place on BUV395) to better resolve the T cells positive for HLA-DR. Due to this, HLA-DR on MNPs had excessively bright expression, and despite low potential, there was a confounding amount of SSE into B610. However, this is also likely autofluorescent macrophages. Advantageously, the low HLA-DR expression on T cells allowed us to gate below the HLA-DR^high^ cells spilling into B610 (gating strategy). When possible, a CD3^+^ magnetic selection is ideal to avoid such issues. There is potential SSE from BV605 into V710 (PD1), but we found no confounding issues on gated CD3^+^ T cells.

The panel was initially designed with a three-marker dump channel. CD14 and CD11c identify mononuclear phagocytes, which would be removed via a dump^+^CD3^-^ gating strategy. We also included CD94 in this channel to identify CD94-expressing T cells in the Dump^+^CD3^+^ channel. However, both CD11c and CD14 have been found on subsets of T cells in activated (DOI: 10.1371/journal.pone.0154253) and tissue settings (DOI: 10.1038/s41586-022-05645-6) respectively. We confirm that CD14 and CD11c are not entirely necessary to improve the purity of lymphocytes, as a second and tighter FSC-A/SSC-A gate on lymphocytes removes these cells (Online Figure 5). With identification of CD94^+^ T cells being of greater interest in anogenital tissues, CD11c and CD14 were removed, leaving CD94 to be placed on AF700, a relatively dim fluorophore. We found the brightness of this fluorophore to be sufficient to resolve CD94^+^ T cells. Minor SSE into R670 (CCR10) and Y820 (CD127) were not an issue due to low expression of CD94. CD123 is expressed on plasmacytoid dendritic cells (pDCs) that infiltrate inflamed tissues. CD123 was included in the panel to assess whether tissues were inflamed (HLA-DR^+^CD123^+^; gating strategy). PE-Cy5 has relatively concerning potential SSE into B710 (CD69) and B820 (CD45) as well as the R670 (CCR10) and R710 (CD94) channels. We placed CD123 on PE-Cy7 as pDCs would be detected within the CD3^-^HLA-DR^high^ population and thus their identification will not be hindered by consequences of SSE from NFB610 (CD3) and APC (CCR10).

There are three major subset categories to consider in this panel: Tissue resident (CD69, CCR5, PD1), Memory (CD28, CD27, CCR7), and Effector/Regulatory (CCR10, CD161, CCR6, TIGIT, CD39) T cells. CD69 identifies tissue resident cells. However, CD69 resolution can be difficult to achieve as it is a secondary marker. BB700 was chosen because it is bright and able to resolve CD69^+^ from CD69^-^ with assistance from CD103 co-expression (gating strategy). SSE from BB700 may affect R670 (CCR10), R710 (CD94), V677 (CCR6) and V710 (PD1). CD69 as a secondary marker allows for minimal SSE by choosing a suboptimal voltage via the stain index while maintaining adequate visual separation (Online Figure 6; see below on optimising voltages). PD-1, an inhibitory receptor, is found on TRMs. As PD1 has a secondary/tertiary expression it was placed on BV711. Potential SSE from BV711 were R670 (CCR10), U740 (CD154) and V820 (CD27), but no issues were found.

Some **chemokine receptors** are used to identify subsets of effector T cells (CCR10 and CCR6) while others are considered as tissue residency markers (CCR5). Chemokine receptors have variable expressions across tissue and blood (DOI: 10.1016/j.immuni.2016.07.007; Online Figure 2), and are secondary/tertiary markers. Therefore, we assigned all of them to medium/bright fluorophores. CCR5 was placed on VioBright B515. There is no confounding SSE from this channel. CCR10 was placed on APC and positive resolution was obtained. From APC, there was potential for SSE into R710 (CD94) and V677 (CCR6), but not problematic as the expression of CCR10 is low. Potential mild SSE from APC into Y670 (CD123) was not an issue as CD123^+^ cells are gated out using HLA-DR and CD3 (described above). CCR6 was placed on BV650. CCR6 is expressed at low levels in some tissues (DOI: 10.1016/j.immuni.2016.07.007), but positive resolution is possible in tissue (gating strategy). There is potential for mild SSE into R670 (CCR10), R710 (CD94) and V710 (PD1), though no issues were found. However, particular care is taken when assessing CCR10 versus CCR6, as CCR6^+^CCR10^-/+^ define Th17, and CCR6^-^CCR10^+^ define Th22. When possible, a CCR6 FMO is included. Fortunately, both markers are relatively dim and pose a minimal risk of SSE into each other. CD161 and CCR6 are both associated with Th17 cells. CD161 has greater expression and resembles more of a secondary marker. We assigned CD161 to BUV615. There were no potential SSE from BUV615 and BV650 into each other, but care was still taken by accurately determining the minimal titration and voltage required to get adequate resolution. Other potential SSE’s from BUV615 are R670 (CCR10) and Y610 (CD103), though we found no issues.

CD28 and CD27 that define the memory subsets were challenging markers to achieve adequate resolution. CD28 may vary between donors based on many factors such as activation, T cell exhaustion and age. We therefore expected both high and low expressions. Both have secondary marker expressions. First, we chose BV510 for CD28, but it was difficult to resolve a positive population (Online Figure 7). Therefore, we moved CD28 to BUV395 (while CD3 was moved to NFB610). Following titrations and voltage optimisation, a negative population was resolved in the CD8^+^ cells. We assigned a dim/medium fluorophore BV786 to CD27. There is potential SSE from BV786 into U740 (CD154) but found no confounding issues. Similarly, CCR7, is not expected to be highly expressed in most tissues (compared to blood) as it is a lymph node homing marker. This made determining appropriate resolution difficult. We chose a medium-bright marker (BV421) for CCR7 and found we could identify CCR7^+^ T cells (gating strategy). There were no major SSE identified.

CD39 and TIGIT define a regulatory function in T cells. We expect that the co-expression of these markers be biologically relevant, and therefore, particular care was taken in choosing appropriate fluorophores to limit spillover into each other. CD39 is a secondary marker but has relatively bright expression. Conjugated to BV480, a clear resolution was achieved. TIGIT is a secondary marker and we assigned it to PE. There was no confounding SSE found between BV480 and PE. There is potential SSE from PE into Y610 (CD103), but no issues were identified.

Expression of activation markers (CD38, CD154, HLA-DQ) will vary depending on whether the tissue is inflamed or healthy, and whether the cells are activated/stimulated or not. CD38 is an early activation marker lowly expressed on resting T cells, and more highly expressed on CD3^-^ plasma cells. We assigned it to the relatively bright APC-Fire810. Potential SSE may occur into B820 (CD45), U820 (CD4), V820 (CD27) and Y820 (CD127). Due to its low expressions on tissue T cells, no problems were identified. However, APC Fire-810 is high on our FMO priority list. CD154 provides costimulatory signals to T cells to initiate proliferation and cytokine production. To our knowledge, CD154 has not been investigated on the surface of TRMs. However, it was differentially expressed in a single cell RNA sequencing dataset of resting CD4^+^ T cells (DOI: 10.1038/s41467-021-22164-6), and therefore we included it in our panel. In resting tissue, CD154 was not found brightly expressed, and thus we assigned it to BUV737 to ensure we captured any low expression. There were no substantial concerns of SSE from the BUV737 channel. Lastly, we included HLA-DQ, an MHC class II heterodimer primarily found on MNPs. It has not been widely explored on the surface of T cells, but it was differentially expressed across multiple single-cell RNA-sequencing datasets (Online Figure 8). We found it dimly expressed on T cells that are HLA-DR^high^. Potential SSE from BUV563 are U379 (CD28), U610 (CD161), Y586 (TIGIT) and Y610 (CD103). However, the low expression of HLA-DR on T cells minimised the SSE concerns. We had one notable concern of the potential SSE of HLA-DR BV650 into U586 (HLA-DQ), as its dual expression is important. However, the lower expression of HLA-DR on T cells mitigates this issue.

**Titrations and voltage optimisation**

We have unique access to a broad range of human anogenital tissues to investigate tissue immune cell properties. Once the panel was designed and the antibody clones and enzymes have been optimised, the markers were individually titrated, and the voltages optimised (Online Figure 9). However, there are some challenges in optimising a tissue panel compared to a blood panel: i) the marker densities and proportions in tissue, between different tissue types, and whether tissues are healthy or inflamed may vary as is the case with CCR7, CD69, CD27 and many activation markers, ii) the number of cells that can be isolated from tissues are considerably lower than those isolated from blood, such as the epidermis of smaller-sized genital tissues will only yield 1x10^6^ cells in total, iii) there is a sparsity of available human tissue for research compared to blood.

To use this panel across multiple tissues and tissue types, we have first optimised each marker on the tissues where markers are known to be expressed brightly or densely. For example, we have previously found CD27 expression to be absent in colorectal tissues (unpublished data), and therefore, we titrated this marker on a skin-type tissue. Secondly, to mitigate the limitation of doing antibody titrations over a large range of titres, we used a two-step approach. First we titrated antibodies over a larger range on PBMCs (4-5 titres; Online Figure 9A), then tested over a smaller range on tissues (2-4 titres; Online Figure 9B). Thirdly, because this panel is targeted towards tissue-specific subsets, some markers are simply not expressed in PBMCs, such as CD103. Therefore, we’ve titrated over a short range only on cells isolated from tissues. Lastly, for activation markers, PBMCs were treated with Phytohaemagglutinin (PHA) (Sigma-Aldrich, 5μg per 2 x 10^6^ cells/mL) + IL-2 (Peprotech, Israel, 150U per 2 x 10^6^ cells/mL) for 3 days prior to staining (Online Figure 9A; denoted by *****). The optimal titres for each antibody are outlined in red boxes.

After determining the optimal titre, tissue cells were stained with each marker on its own and acquired on the FACSymphony at different voltages (25 Volt increments, Online Figure 6). Both the optimal titre and optimal voltage were determined using the following stain index equation:

$$\frac{MFI(+)-MFI(-)}{2 * SD(-)}$$

High voltages increase the possibility of causing SSE, and therefore, *suboptimal* voltages were chosen based on the visually identifying separation. For example, the voltage 675 for CD69 had the greatest stain index (blue asterisk), but a lower voltage (600) was chosen (red asterisk) as separation was clearly achieved (Online Figure 6).

**Online Table 1:** BD FACSymphony A5 Machine Configuration

| Laser | Wavelength (nm) | Laser Type | Laser Power (mW) | Dichroic filter (nm) | Notch filter (nm) | Bandpass Filter (nm) | Spectral Range | Assigned Fluorochrome |
| --- | --- | --- | --- | --- | --- | --- | --- | --- |
| UV | 355 |  | 100 |  |  | 379/28 BP | 365-393 | BUV395 |
|  |  |  |  | 410 LP |  | 450/50 BP | 425-450 | FVS UV440 |
|  |  | Solid State OBIS 637 LX |  | 450 LP |  | 515/30 BP | 500-530 | BUV496 |
|  |  |  |  | 570 LP | 594 | 586/15 BP | 578.5-593.5 | BUV563 |
|  |  |  |  | 600 LP |  | 610/20 BP | 600-620 | BUV615 |
|  |  |  |  | 635 LP |  | 670/30 BP | 655-685 |  |
|  |  |  |  | 685 LP |  | 695/40 BP | 685-710 |  |
|  |  |  |  | 710 LP |  | 740/35 BP | 722.5-757.5 | BUV737 |
|  |  |  |  | 770 LP |  | 780/60 BP | 770-800 |  |
|  |  |  |  | 800 LP | 785 | 820/60 BP | 800-850 | BUV805 |
| Violet | 406 |  | 200 |  |  | 405/10 BP | 400-410 | vSSC |
|  |  |  |  | 410 LP |  | 427/25 BP | 415.5-438.5 | BV421 |
|  |  | Solid State OBIS 405 LX |  | 450 LP |  | 474/25 BP | 462.5-485.5 | BV480 |
|  |  |  |  | 505 LP |  | 525/50 BP | 505-550 |  |
|  |  |  |  | 550 LP | 594 | 586/15 BP | 578.5-593.5 |  |
|  |  |  |  | 600 LP |  | 610/20 BP | 600-620 | BV605 |
|  |  |  |  | 635 LP |  | 677/20 BP | 667-685 | BV650 |
|  |  |  |  | 685 LP |  | 710/50 BP | 685-735 | BV711 |
|  |  |  |  | 735 LP |  | 750/30 BP | 735-765 |  |
|  |  |  |  | 770 LP | 785 | 780/60 BP | 770-800 |  |
|  |  |  |  | 800 LP |  | 820/60 BP | 800+850 | BV786 |
| Blue | 488 |  | 200 |  |  | 488/10 BP | 483-493 | SSC |
|  |  |  |  | 505 LP |  | 515/30 BP | 505-530 | VioB515 |
|  |  | Solid State Sapphire 488 LP |  | 600 LP | 594 | 610/20 BP | 600-620 | NovaFluor B610 |
|  |  |  |  | 635 LP |  | 670/30 BP | 655-685 |  |
|  |  |  |  | 685 LP |  | 710/50 BP | 685-735 | BB700 |
|  |  |  |  | 735 LP |  | 750/30 BP | 735-765 |  |
|  |  |  |  | 770 LP | 785 | 780/60 BP | 770-800 |  |
|  |  |  |  | 800 LP |  | 820/60 BP | 800-850 | BB820-P |
| Yellow | 561 |  | 150 | 570 LP | 594 | 586/15 BP | 578.5-593.5 | PE |
|  |  | Solid State OBIS 561 LS |  | 600 LP |  | 610/20 BP | 600-620 | PE Vio615 |
|  |  |  |  | 635 LP |  | 670/30 BP | 655-685 | PE-CY5 |
|  |  |  |  | 685 LP |  | 710/50 BP | 685-735 |  |
|  |  |  |  | 750 LP | 785 | 780/60 BP | 750-800 |  |
|  |  |  |  | 800 LP |  | 820/60 BP | 800-850 | PE-CY7 |
| Orange | 594 | Solid State OBIS 594 LS | 100 | 610 LP |  | 616/23 BP | 610-627.5 |  |
|  |  |  |  | 635 LP |  | 660/20 BP | 650-670 |  |
| Red | 637 | Solid State OBIS 637 LX | 140 | 665 LP |  | 670/30 BP | 665-685 | APC |
|  |  |  |  | 685 LP |  | 710/40 BP | 690-730 | AF700 |
|  |  |  |  | 750 LP | 785 | 780/60 BP | 750-800 |  |
|  |  |  |  | 800 LP |  | 820/60 BP | 800-850 | APC-Fire 810 |
| Indigo/NIR | 446/779 | Solid State OBIS 785 | 100 | 450 LP |  | 470/15 BP | 462.5-477.5 |  |
|  |  |  |  | 500 LP |  | 515/30 BP | 500-530 |  |
|  |  |  |  | 600 LP |  | 605/40 BP | 600-625 |  |
|  |  |  |  | 635 LP |  | 660/20 BP | 650-670 |  |
|  |  |  |  | 800 LP |  | 820/60 BP | 800-850 |  |

**Online Table 2:** Commercial reagents used in OMIP

| **Specificity** | **Fluorochrome** | **Clone** | **Catalog. Number** | **Manufacturer** | **Dilution** |
| --- | --- | --- | --- | --- | --- |
| CD28 | BUV395 | CD28.2 | 740308 | BD | 1:50 |
| Viability | FVS UV440 | - | 566332 | BD | 1:2000 |
| CD8 | BUV496 | SK1 | 741199 | BD | 1:100 |
| HLA-DQ | BUV563 | TU169 | 748563 | BD | 1:50 |
| CD161 | BUV615 | HP-3G10 | 751374 | BD | 1:20 |
| CD154 | BUV737^∇^ | TRAP-1 | 748983 | BD | 1:20 |
| CD4 | BUV805 | OKT4 | 750976 | BD | 1:25 |
| CCR7 * | BV421 | 2-L1-A | 566743 | BD | 1:33 |
| CD39 | BV480 | TU66 | 746454 | BD | 1:33 |
| HLA-DR | BV605 | G46-6 | 562845 | BD | 1:50 |
| CCR6 * | BV650^∇^ | 11A9 | 563922 | BD | 1:20 |
| PD1 | BV711^∇^ | EH12.1 | 352328 | BD | 1:50 |
| CD27 | BV786^∇^ | L127 | 563327 | BD | 1:33 |
| CCR5 * | VioBright B515 | REA245 | 130-120-57 | Miltenyi Biotec | 1:20 |
| CD3 | NF Blue 610-70S | UCHT1 | H002T03B06 | ThermoFisher | 1:25 |
| CD69 | BB700 | FN50 | 747520 | BD | 1:20 |
| CD45 | BB790-P | HI30 | Custom | BD | 1:200 |
| TIGIT | PE | A15153G | 372704 | BioLegend | 1:20 |
| CD123 | PE-Cy5 | 6H6 | 306008 | BioLegend | 1:200 |
| CD103 | PE-Vio615 | REA803 | 130-111-837 | Miltenyi Biotec | 1:20 |
| CD127 | PE-Cy7 | R34.34 | A64618 | Beckman Coulter | 1:20 |
| CCR10 * | APC | 1B5 | 564771 | BD | 1:33 |
| CD94 | AF700 | KLRD1 | Ab272350 | Abcam | 1:100 |
| CD38 | APC-Fire810^∇^ | HIT2 | 303550 | BioLegend | 1:13 |

* Stained 15 minutes prior to others

∇ High FMO priority^[[1]](#footnote-1)^

Online Table 3: Experiment iterations of panel development

| **Channel** | **Fluorochrome** | **Iteration 1** | **Iteration 2** |  |
| --- | --- | --- | --- | --- |
| U379 | BUV395 | CD3 | CD28 | Changed |
| U450 | UV440 | Viability | Viability | Removed |
| U515 | BUV496 | CD8 | CD8 | Unused channel |
| U586 | BUV563 | HLA-DQ | HLA-DQ |  |
| U610 | BUV615 | CD161 | CD161 |  |
| U740 | BUV737 | CD154 | CD154 |  |
| U820 | BUV805 | CD4 | CD4 |  |
| V427 | BV421 | CCR7 | CCR7 |  |
| V474 | BV480 | CD39 | CD39 |  |
| V525 | BV510 | CD28 |  |  |
| V610 | BV605 | HLA-DR | HLA-DR |  |
| V677 | BV650 | CCR6 | CCR6 |  |
| V710 | BV711 | PD-1 | PD-1 |  |
| V820 | BV786 | CD27 | CD27 |  |
| B515 | VioB515 | CCR5 | CCR5 |  |
| B610 | NFB610 |  | CD3 |  |
| B710 | BB700 | CD69 | CD69 |  |
| B820 | BB790 | CD45 | CD45 |  |
| Y586 | PE | TIGIT | TIGIT |  |
| Y610 | PE Vio 615 | CXCR6 | CD103 |  |
| Y710 | PE-CY5 | CD123 | CD123 |  |
| Y820 | PE-CY7 | CD127  (HIL-7R-M21) | CD127 (R34.34) |  |
| R670 | APC | CCR10 | CCR10 |  |
| R730 | AF700 | CD94, CD14, CD11c | CD94 |  |
| R780 | APC-Fire750 | CD103 |  |  |
| R820 | APC-Fire810 | CD38 | CD38 |  |

**Online Table 5:** Antibodies used but not included

| **Specificity** | **Fluorochrome** | **Clone** | **Reason** |
| --- | --- | --- | --- |
| CD4 | BV785 | RPA-T4 | Clone testing – sup-optimal resolution due to cleavage |
| CD8 | PerCP Cy5.5 | RPA-T8 | Clone testing – sup-optimal resolution due to cleavage |
| CCR7 | BV421 | 150503 | Clone testing – sub-optimal resolution possibly due to enzymatic cleavage |
| CD69 | Pe-Cy7 | L78 | Clone testing – sup-optimal resolution due to cleavage |
| CD127 | PE-Cy7 | HIL-7R-M21 | Clone testing – sub-optimal resolution due to cleavage |
| CD103 | APC-Fire750 | Ber-ACT8 | Clone testing – sub-optimal resolution due to cleavage |
| CD27 | BV510 | O323 | Clone – testing – sub-optimal resolution, possibly to susceptibility to enzyme cleavage |
| CD161 | BV510 | DX12 | Clone testing – sub-optimal resolution, possibly due to susceptibility to enzyme cleavage |
| CD28 | BV510 | CD28.2 | Sub-optimal fluorochrome placement; resolved by moving to BUV395 |
| CD3 | BUV395 | UCHT1 | Panel reshuffle; moved to NovaFluor B610 |
| CXCR6 | PE-Dazzle594 | K041E5 | Panel reshuffle; removed |
| CD14 | AF700 | M5E2 | Not required in exclusion channel |
| CD11c | AF700 | B-ly6 | Not required in exclusion channel |

**Online Table 6:** Compensation reagents used. For each compensation control, 50µL of positive and 50µL of negative beads were combined and washed (350 x *g* for 5 minutes) in Stain Buffer (1% FBS (v/v), 2mM EDTA, 0.1% sodium azide (w/v) in PBS). Supernatant was removed and beads were resuspended in 100µL of Stain Buffer and stained for 15 minutes at 25˚C. Beads were then washed twice using Stain Buffer. Dilution is shown as antibody volume in µL/100µL.

| **Specificity** | **Fluorochrome** | **Compensation Bead** | **µL** |
| --- | --- | --- | --- |
| CD28 | BUV395 | BD CompBeads Plus Anti-Mouse Ig (Normal) | 1 |
| CD8 | BUV496 |  | 1 |
| HLA-DQ | BUV563 |  | 1 |
| CD161 | BUV615 |  | 1 |
| CD154 | BUV737 |  | 1 |
| CD4 | BUV805 |  | 1 |
| CCR7 | BV421 |  | 0.9 |
| CD39 | BV480 |  | 1.2 |
| HLA-DR | BV605 |  | 1 |
| CCR6 | BV650 |  | 1 |
| PD1 | BV711 |  | 1 |
| CD27 | BV786 |  | 1.6 |
| CD3 | NF Blue 610-70S |  | 1 |
| CD69 | BB700 |  | 1.5 |
| CD45 | BB790-P |  | 1 |
| TIGIT | PE |  | 1 |
| CD123 | PE-Cy5 |  | 1 |
| CD127 | PE-Cy7 |  | 1 |
| CCR10 | APC |  | 1.2 |
| CD94 | AF700 |  | 1.5 |
| CD38 | APC-Fire810 |  | 1 |
| CCR5 | VioBright B515 | MACS Comp Bead Kit, anti-REA | 1 |
| CD103 | PE-Vio615 |  | 1 |
| Viability | FVS UV440 | Arc Amine Reactive Compensation Bead Kit | 1 |

**Tissue Digestion and Staining Protocol**

**Ethics statement**

This study was approved by the Ethics Committee for Western Sydney Local Health District,

approval no. 4192–2019/ETH01894. Written informed consent was received from participants

prior to inclusion in this study.

**Materials**

- Dulbecco’s Phosphate Buffered Saline Mg^++^ / Ca^++^ free (DPBS) (Lonza, Switzerland)
- DNase I (Worthington Industries, New Jersey, USA)
- Foetal Bovine Serum (FBS) (Sigma-Aldrich, Missouri, USA)
- Dithiothreitol (DTT) (Sigma-Aldrich)
- EDTA (Sigma-Aldrich)
- RPMI 1640 (Lonza)
- Brilliant Stain Buffer Plus (BD Biosciences, San Jose, CA)
- MACSmix Tube Rotator (Miltenyi Biotec, California, USA)
- 100 μM cell strainer (Greiner Bio-One, North Carolina, USA)
- Collagenase Type IV (Worthington Industries, New Jersey, USA)
- Ficoll-Paque PLUS (GE, Healthcare, Illinois, USA)
- BD Cytofix (BD, Biosciences)
- Dispase (Neutral Protease NPRO2) (Worthington Industries)
- Skin graft knife (Swann-Morton, Sheffield, United Kingdom)
- Skin graft mesher (Zimmer Bionet, Warsaw, IN, USA)
- Gentamicin (Gibco, Waltham, MA, USA)

**Solutions:**

- **RF10** - 10% FBS in RPMI 1640
- **Stain Buffer** – 1% FBS, 2mM EDTA and 0.1% Sodium azide in DPBS
- **Epithelial Strip** **Solution**– 0.3% DTT and 2mM EDTA in RF10
- **Dispase Solution** – 1U/mL Dispase in RPMI with 50ug/mL Gentamicin and filter sterilised with 0.22µm filter
- **Collagenase Solution** – 200U/mL Collagenase IV with 100U/mL DNase I in RPMI

**Intestinal tissue digestion**

1. Fat and muscularis was removed using surgical scissors and a scalpel.
2. Tissue was diced into ~2cm pieces.
3. Tissue was incubated in a 50mL Falcon tube containing 20mL of the Epithelial Strip Solution for 15 minutes at 37˚C.
4. The supernatant was discarded using a tea strainer and tissue was placed back into the tube.
5. Steps 3 and 4 were repeated.
6. Tissue was rinsed with 10mL of DPBS and strained through the tea strainer.
7. Tissue was incubated in 20mL Collagenase Solution (pre-warmed to 37 ˚C) on the MACSmix Tube Rotator for 30 minutes at 37˚C.
8. Supernatant was collected using a tea strainer.
9. Steps 7 and 8 were repeated.
10. Cells collected from Step 8 were passed through a 100µm cell strainer and resuspended in DPBS.
11. Cells were centrifuged for 5 minutes at 450 x *g*.
12. The supernatant was poured off and the wash cycle was repeated.
13. Cells were resuspended in 10mL of RPMI and left on ice.
14. Steps 10 – 13 were repeated with cells collected at step 9.
15. The two collections of cells were combined into a total of 35mL RPMI.
16. Cells were underlaid with 15mL Ficoll-Paque PLUS and centrifuged at 450 x *g* for 20 minutes with no brake.
17. Cells were harvested from the RPMI-Ficoll interface.
18. Cells were resuspended in 50mL DPBS and centrifuged for 5 minutes at 350 x *g*.
19. The supernatant was poured off and the wash cycle was repeated.

**Abdominal skin tissue digestion**

1. Abdominal skin was stretched using large forceps and grafted to ~1mm thickness.
2. Skin grafts were processed through a skin graft mesher.
3. Meshed tissue was evenly distributed into 50mL Falcon tubes up to about 20% of its volume.
4. Tissue was incubated in 35mL of the Dispase Solution on a rotator at 4˚C overnight.
5. The following morning, tubes were placed in 37˚C water bath for 15 minutes.
6. Tissue was dunked in a 50mL falcon tube containing 40mL DPBS and the epidermis was mechanically separated from the dermis using forceps.
7. Dermal and Epidermal tissues were separated and placed in separate 50mL Falcon tubes.
8. Dermal tissue was diced into small, 1 cm squares and distributed amongst 50mL Falcon tubes, exceeding no higher than the 5mL mark.
9. Epidermal tissue was collected into a 50mL Falcon tube and chopped into small pieces using scissors.
10. Cells were incubated in 20mL of the Collagenase Solution in a 50mL Falcon tube, initially placed in a 37˚C waterbath for 10 mins to get to solution up to temperature quickly, then moved to a rotator for up to 110 minutes. If no rotator is available, cells can be placed in a waterbath and shaken manually every 10 mins.

**Note**: Incubations may be stopped nearer 90 minutes if the solution becomes overtly cloudy and most tissue is digested, indicating that cells have been liberated from the tissue. If the cells remain too long in the solution, viability and maturation are affected (DOI: 10.1189/jib.4A1116-496R).

1. Cells were collected by passing supernatant through a tea strainer and through a 100µm cell strainer.
2. Cells were washed 3 x in 50mL of DPBS at 350 x *g* for 5 minutes. Supernatants were poured off and resuspended in fresh media each time.
3. Epidermal cells were resuspended in 35mL of DPBS and undergo steps 16-19 from Intestinal tissue digestion.

**Genital tissue digestion**

1. Genital tissues such as Vagina and Labia are smaller, and therefore, underlying submucosa were removed using scissors or a scalpel, and forceps.
2. A scalpel was then used to create a mesh pattern in the tissue.
3. Steps 4-13 from Abdomen skin tissue digestion were then undertaken.

**Note**: Epidermal cell yields are much smaller and therefore step 13 was only performed if an adequate cell pellet was achieved by step 13. The cells were resuspended in 10mL DPBS and underlaid with 5mL Ficoll-Paque PLUS.

**Staining Protocol**

1. 1.0 - 2.5 x 10^6^ isolated cells from tissue were collected in a 5mL FACS tube.
2. Cells in 100µL were stained with Fixable Viability Stain UV440 for 30 minutes at 4˚C.
3. Cells were washed in 1 mL staining buffer and centrifuged at 350 x *g* for 5 minutes.
4. The supernatant was removed, and cells resuspended in 50µL were stained with a chemokine receptor antibody cocktail: 10µL Brilliant Buffer PLUS with chemokine receptor antibodies for 15 minutes at room temperature (noted in Online Table 2).
5. The rest of the antibodies were added to the tube, made up to a final volume of 100µL using Stain Buffer, vortexed, and incubated for 30 minutes at room temperature.
6. Cells were washed in 1 mL staining buffer and centrifuged at 350 x g for 5 minutes.
7. Supernatant was removed and step 6 was repeated.

**Optional steps – all samples included in this OMIP were fixed prior to acquisition, this is dependent on the downstream application of the panel.**

O1 Cells were resuspended in 150µL BD Cytofix and incubated for 15 minutes at 4˚C

O2 Step 6 and 7 were repeated.

1. Cells were resuspended in 100µL of Stain Buffer and acquired on the FACSymphony A5

1. Low cell yields from tissue can restrict a full list of appropriate FMOs every time. Therefore, we choose certain markers that are priority for FMO staining when cell yields permit. [↑](#footnote-ref-1)
