# Supplementary material for: OMIP‐096: A 24‐color flow cytometry panel to identify and characterize CD4+ and CD8+ tissue‐resident T cells in human skin, intestinal, and type II mucosal tissue
Source: Cytometry A. 2023 Sep 29;103(11):851–6. doi: 10.1002/cyto.a.24782 (PMC10953338; doi:10.1002/cyto.a.24782)
Supplement: Supplementary file 3 — MIFlowCyt MIFlowCyt item checklist. [file CYTO-103-851-s001.doc]

**Cytometry Part A**

**Author Checklist: MIFlowCyt-Compliant Items**

| **Requirement** | **Please Include Requested Information** |
| --- | --- |
| 1.1. Purpose | This panel was developed and optimised for the measurement of tissue resident T cells, isolated from human tissue, including intestinal and abdominal skin tissue, type II mucosae. |
| 1.2. Keywords | Flow cytometry, TRMs, tissue-resident T cells, CD4, CD8, human tissue, enzymatic digestion, flow cytometry |
| 1.3. Experiment variables | Frequency of cell types and their activation status |
| 1.4. Organization name and address | The Westmead Institute for Medical Research, Westmead, NSW, Australia,  The University of Sydney, NSW, Australia |
| 1.5. Primary contact name and email address | Kirstie M Bertram, Kirstie.bertram@sydney.edu.au |
| 1.6. Date or time period of experiment | 22/Feb/2022 |
| 1.7. Conclusions | Development, validation, and cross-validation of 24-colour flow cytometry |
| 1.8. Quality control measures | Verification of antibody clones and testing of enzyme-blend cleavage  Fluorescence Minus One controls  Multiple human tissue types  BD CST beads for instrument QC prior to each experiment |
| 2.1.1.1. (2.1.2.1., 2.1.3.1.) Sample description | Cells isolated from human intestinal tissue, human genital skin |
| 2.1.1.2. Biological sample source description | Human, tissue discarded from surgery |
| 2.1.1.3. Biological sample source organism description | Human Adults |
| 2.1.2.2. Environmental sample location | N/A |
| 2.3. Sample treatment description | See appendix |
| 2.4. Fluorescence reagent(s) description |  |
| 3.1. Instrument manufacturer | Becton Dickinson (BD) |
| 3.2. Instrument model | FACSymphony A5 |
| 3.3. Instrument configuration and settings | 8 lasers, 46 detectors.  355nm, 100mW (10)  406nm, 200mW (10+SSC)  446nm, 75mW (4)  488nm, 150mW (7+FSC/SSC)  561nm, 150mW (6)  594nm, 100mW (2)  637nm, 140mW (4)  779nm, 100mW (1)  (See Online Table 1 – Instrument Configuration) |
| 4.1. List-mode data files | *We recommend all authors to submit their data files to [http://flowrepository.org](http://flowrepository.org/) and to make them available for the peer-review process. If you have done so, please let us know by inserting the following codes (replace the red text):  1) The link for peer-review process:  <https://flowrepository.org/id/RvFr4UZvo12tjNoD8hl8SBMkcaHHmbhpZJCIMn2aaj33hMlW6KsugeRTag1WLyPx>.This link will only be shared with reviewers of your manuscript. |
| 4.2. Compensation description | Single-stained controls using compensation beads from BD Biosciences, Miltenyi Biotec, and Thermofisher. Compensation was performed using the compensation wizard in FlowJo 10.8.  (See Online Table 6 – Compensation reagents) |
| 4.3. Data transformation details | Biexponential transformation (Custom transformation and Manually Specify transformation options) in FlowJo 10.8. |
| 4.4.1. Gate description | Hierarchical gating to identify lineages and then comparative histograms to visualize activation markers. |
| 4.4.2. Gate statistics | See Figure 1 |
| 4.4.3. Gate boundaries | See Figure 1 |

**Notes**

Feel free to use more space than allocated.

You can embed graphics/figures in this document, if needed.

Please make sure to save the document in Microsoft Word version 2003 or older, before uploading to ScholarOne Manuscripts. When uploading this checklist to ScholarOne Manuscripts, please choose the “Supplementary Material for Review” category.

Please note that if your paper is accepted, the checklist will be published as an Online Supporting Information.

For any questions, please contact the Cytometry Part A editorial office at [Cytometrya@wiley.com](mailto:Cytometrya@wiley.com).
